# Production of Recombinant African Swine Fever Viruses: Speeding Up the Process

**DOI:** 10.3390/v12060615

**Published:** 2020-06-05

**Authors:** Anusyah Rathakrishnan, Katy Moffat, Ana Luisa Reis, Linda K. Dixon

**Affiliations:** The Pirbright Institute, Ash Road, Pirbright, Surrey GU24 0NF, UK; anusyah.rathakrishnan@pirbright.ac.uk (A.R.); Kathryn.Moffat@pirbright.ac.uk (K.M.); ana.reis@pirbright.ac.uk (A.L.R.)

**Keywords:** ASFV, FACS, recombinant virus, EP153R, EP402R, DP148R

## Abstract

African swine fever (ASF) is a devastating disease in pigs, with no vaccines for control. The genetic manipulation of African swine fever virus (ASFV) is often tedious and time consuming. Here, we describe a method to manipulate the virus genome to produce gene deletion viruses in a much-reduced time. This method combines the conventional homologous recombination with fluorescent-activated cells sorting (FACS), to isolate and purify viruses expressing fluorescent reporter genes. With three rounds of single cell isolation via FACS and two rounds of limiting dilution, we deleted two additional genes, EP153R and EP402R, from Benin 97/1 ASFV lacking the DP148R gene. By combining different fluorescent markers, this method has the potential to greatly facilitate studies on understanding ASFV gene functions and develop candidate live-attenuated vaccines.

## 1. Introduction

African swine fever virus (ASFV), the sole member of the family *Asfaviridae*, causes African swine fever (ASF) disease in European wild boar and domestic pigs. With lethality rates reaching 100% after infection with virulent ASFV strains, the acute form of disease is characterized by fever, loss of appetite, lethargy and haemorrhage. The less virulent strains can lead to mild clinical signs, subacute forms of disease, or even chronic infections. Warthogs and bush pigs develop mild or no clinical signs, even when infected with highly virulent isolates and, thus, they can act as reservoirs for infection [1].

ASF is endemic in many sub-Saharan African countries. Since 2007, countries in Eastern Europe, and more recently, at least 11 countries in Asia have reported numerous ASF outbreaks. ASF has a huge socio-economic impact, as pigs are important sources of protein. China is among the world’s largest producers and consumers of pork and pork-related products. ASFV infection in these countries has thus led to destabilization of the pork industry throughout the world [2].

ASFV is either transmitted directly between animals or indirectly via feeding of infected meat or by products, or through fomites. Therefore, controlling ASF relies heavily on zoning or restricting movement of animals, the immediate culling of pigs in infected areas and rigorous surveillance. No vaccines nor antivirals exist for ASF, mainly due to the gap in knowledge regarding ASFV, including (i) the unknown functions of at least half of the encoded 150–167 proteins; (ii) the absence of correlates of protection and a general poor understanding of protective immunity; and (iii) the need for primary cells to propagate and manipulate the virus [3,4]. 

Rational deletions of ASFV virulence genes or immune response inhibitory genes, is often seen as the fastest route to vaccine development [3]. Serial gene deletions allow optimization of safety and efficacy of recombinant live attenuated vaccines (LAVs). However, producing these viruses with multiple gene deletions remains a major bottleneck since deleting a single gene can take up to 3–4 months. Conventionally, genetic manipulation of ASFV for producing LAV candidates or for gene function studies have relied on using homologous recombination, whereby ASFV genes are replaced with a reporter gene including β-galactosidase (β-Gal) or β-glucuronidase (GUS) [5,6,7,8,9] or more recently, fluorescent reporter genes [10,11,12,13,14]. Using the Cre/ loxP recombination system, sequential deletion of multiple ASFV genes has been performed to allow the same reporter genes to be used several times. However, this requires additional steps and therefore extended production time [15]. Homologous recombination events are rare, and to improve this initial efficiency, Borca and colleagues applied the CRISPR/Cas9 system to facilitate gene deletion [16]. Nevertheless, the subsequent steps involve multiple rounds of limiting dilution or plaque purification to obtain recombinant viruses, without contamination from the parental virus. This makes the overall production of gene deleted ASFV a long process. 

Here, we describe a method to speed up the production of recombinant ASFV by taking advantage of fluorescent reporter markers and single cell isolation fluorescence-activated cell sorting (FACS). The time to make deletions has been shortened from 3–4 months to 1–1.5 months using this method. This, combined with the ability to use at least three different fluorescent reporter genes simultaneously, can positively contribute to the production of recombinant ASFV that can be used as vaccine candidates and to study viral gene functions.

## 2. Materials and Methods 

### 2.1. Cells and Viruses

Spontaneously immortalized wild boar cell line, WSL-R was kindly provided by Dr. Walter Fuchs (Friedrich-Loeffler-Institut, Greifswald—Insel Riems, Germany) and grown in ZB28 medium (50% Gibco^TM^ Ham’s F12 Nutrient Mix medium (Thermo Fisher Scientific, Waltham, MA, USA) + 50% Gibco^TM^ IMDM) supplemented with 10% fetal bovine serum (FBS) (Life Science Production, Bedford, UK), 1% Gibco^TM^ Penicillin-Streptomycin, and 1% Gibco^TM^ Glutamine. Primary porcine bone marrow cells (PBMs) were derived from the long leg bones of 4-week-old outbred pigs, and were maintained in Gibco^TM^ EBSS supplemented with 10% porcine serum (Biosera, Nuaille, France), 1% Penicillin-Streptomycin and 1% Gibco^TM^ HEPES. Additionally, PBMs were centrifuged at 1000 *g* for 30 min on a density gradient at 1.083 g/mL in Histopaque (Sigma-Aldrich, Dorset, UK). The buffy coat was collected, red blood cells lysed with 1X RBC Lysis Buffer (BioLegend, San Diego, CA, USA) and washed with PBS, to obtain purified PBMs that could be frozen down in freezing medium constituted of 90% FBS and 10% DMSO (Sigma-Aldrich, Dorset, UK). Thawed purified PBMs were maintained in RPMI 1640 medium, supplemented with Gibco^TM^ Glutamax, 10% FBS, 1% Penicillin-Streptomycin and 100 ng/mL of recombinant porcine macrophage colony-stimulating factor (CSF1) (Roslin Technologies, Midlothian, UK). 

ASFV isolates used in this study include Benin 97/1 (genotype I) [17], Benin∆DP148R [18] and Benin∆EP402R (unpublished data). Propagation and titrations of virus were carried out using PBMs or purified PBMs. Virus titrations were carried out by endpoint dilution assays either via haemadsorption (HAD_50_/mL) [19] or by detection of infected fluorescent cells (TCID_50_/mL). Virus titres were calculated using the Spearman and Kärber algorithm [20].

### 2.2. Production of Recombinant ASFV Benin∆EP153R∆EP402R∆DP148R

We aimed to produce a recombinant ASFV from previously published attenuated Benin∆DP148R by further deleting the genes EP153R and EP402R. We combined the conventional homologous recombination with single cell isolation via FACS to produce a final recombinant Benin∆EP153R∆EP402R∆DP148R.

#### 2.2.1. Transfer Plasmid

**Figure 1:**
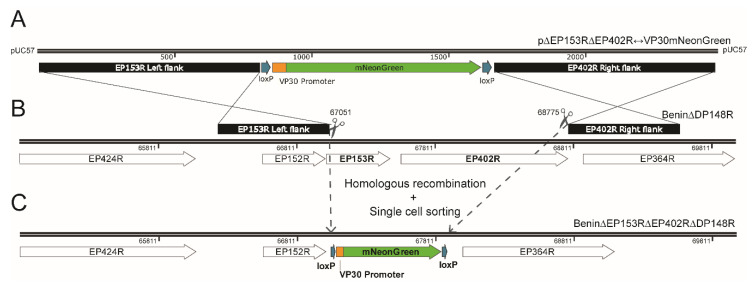
Schematic diagram depicting the generation of recombinant African swine fever (ASFV) isolate lacking genes EP153R and EP402R using homologous recombination and single cell sorting. Homologous recombination between transfer plasmid p∆EP153R∆EP402R↔VP30mNeonGreen (**A**) and parental Benin∆DP148R (**B**) removed genes EP153R and EP402R (genome position: 67,051 to 68,775) from parental virus and replaced with a VP30mNeonGreen reporter gene cassette from the transfer plasmid to generate Benin∆EP153R∆EP402R∆DP148R (**C**). Gene maps were copied from SnapGene® Viewer software (GSL Biotech, San Diego, CA, USA; available at snapgene.com) and modified on Adobe^®^ Illustrator^®^ CS6 (Adobe Systems Inc., San Jose, CA, USA).

A donor/ transfer plasmid containing: (i) the mNeonGreen fluorescent reporter gene [21], under the control of the ASFV VP30 (CP204L) promoter [22]; (ii) approximately 0.8 kbp left flanking arm of ASFV EP153R; and (iii) 0.8 kbp right flanking region of ASFV EP402R, was synthesized from the Benin 97/1 isolate (GenScript, Leiden, the Netherlands) (Figure 1A). The whole fragment (2478 bp) was cloned in pUC57, using the proprietary CloneEZ^®^ Seamless cloning technology with restriction sites *Eco*RI/ *Hind*III and verified via restriction digestion, vector and target sequencing. Plasmid DNA was transformed in DH5α *E. coli* and was extracted using QIAGEN Plasmid Midi Kit (Qiagen, Hilden, Germany), according to the manufacturer’s instructions. 

#### 2.2.2. Homologous Recombination—Infection/Transfection

WSL-R cells were infected with Benin∆DP148R [18] at the multiplicity of infection (MOI) of 2, and plates were centrifuged at 600 *g* for 1 h. After 3 h of incubation at 37 °C, transfection mixture containing Gibco^TM^ OptiMEM, 500 ng of transfer plasmid (2.2.1) and TransIT-LT1 (Mirus Bio, Madison, WI, USA) was added to the infected cells, and centrifuged again at 600 *g* for an hour. Infected/ transfected cells were incubated at 37 °C, 5% CO_2_ for 48 h.

#### 2.2.3. Single Cell Isolation via FACS

The infected/ transfected cells were harvested and pooled after 48h. The cells were washed and re-suspended at 3 × 10^5^ cells/mL in chilled sterile FACS buffer (CaCl_2_ and MgCl_2_ free—Dulbecco’s PBS supplemented with 5 mM EDTA, 25 mM HEPES and 1% FBS) and passed through a 70 μm cell strainer (BD Biosciences, San Jose, CA, USA) prior to cell sorting. 

For single cell sorting, we used DIVA8 acquisition software with a FACSAria Fusion cell sorter (BD Biosciences) equipped with a 405 nm (violet), 488 nm (blue), 561 nm (yellow green) and 640 nm (red) laser. Specifically, for producing Benin∆EP153R∆EP402R∆DP148R, the mNeonGreen infected-transfected cells were detected with a blue 530/30 BP filter and the violet 450/40 BP signal was collected and used to gate out autofluorescent cells. In sorts where viruses with a red fluorescent protein was used as a reporter, a yellow/green 582/15 BP filter was used to detect the fluorescence. Sorting was done with the 85 μm nozzle at 45 psi and in single cell precision mode. To sterilize the flow cytometer between sorts, 2.3% sodium hypochlorite was run at a flow rate of 5, for 5 min, as recommended by OIE [23]. 

**Figure 2:**
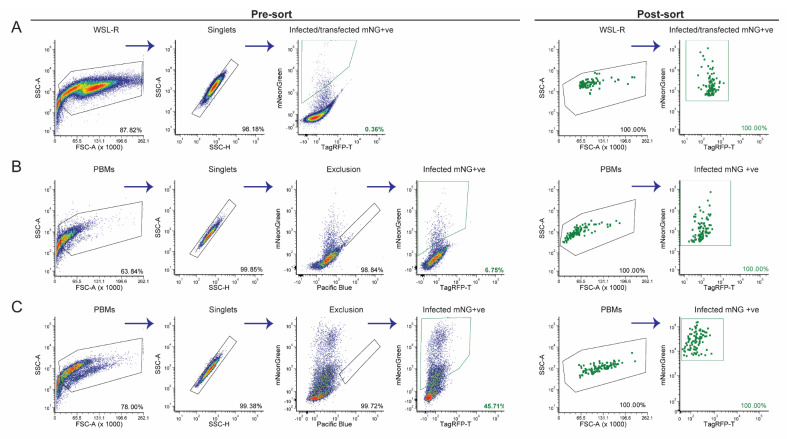
Purification of recombinant Benin∆EP153R∆EP402R∆DP148R via single cell isolation using FACS. The biparametric flow cytometry profiles depicted include two phases. Phase 1—pre-sorting: Panel 1—SSC-A vs FSC-A for total cells; Panel 2—SSC-A vs. SSC-H to obtain singlets; Panel 3—blue BP 530/30 nm-A vs. violet BP 450/40 nm-A to exclude autofluorescence cells, prominently in porcine bone marrow cells (PBMs); Panel 4—blue BP 530/30 nm-A vs. yellow-green (YG) BP 582/15nm-A to capture the mNeonGreen (mNG) positive cell subpopulation. Phase 2—post-sorting: Panel 5—total cells that were single cell sorted into 96-well plate containing purified PBMs; Panel 6—each dot represents an individual mNG positive cell that was sorted. The flow cytometry profiles are shown for (**A**) after the homologous recombination event in WSL-R cells; (**B**) second round of single cell sorting of recombinant ASFV-infected PBMs from a single mNG positive well, monitored under a fluorescent microscope; (**C**) third round of single cell isolation of a single mNG positive well, similar to (B), except the gating for mNG positive subpopulation was more stringent to capture the brighter selection of cells for single cell isolation. The percentages in each panel reflect the percentage of cells within that gate relative to the parent gate.

The FACS gating strategy to identify virus infected-transfected cells is shown in Figure 2. In brief, samples were gated based on cell size/granularity (SSC-A vs. FSC-A) and singlets (SSC-H vs. SSC-A), autofluorescent cells were identified as being dual positive for violet and mNeonGreen and were excluded. Virus infected-transfected cells were then identified as single positive for mNeonGreen. Single color controls were used for compensation and non-infected or non-transfected controls were used to set thresholds (Figures S1 and S2)

The cells were collected at single cell density into individual wells of Thermo Scientific™ Nunc™ MicroWell™ 96-Well microplates (Thermo Fisher Scientific), containing 5 × 10^5^ purified PBMs in 100 μL media. The microplates were then incubated at 37 °C, 5% CO_2_ for 5 days. Cultures were then monitored for expression of mNeonGreen under an inverted fluorescent microscope (Olympus CKX41; Olympus, Tokyo, Japan).

To purify recombinant ASFV, now with additional EP153R and EP402R genes deleted and replaced with reporter gene mNeonGreen, wells in which fluorescent cells were observed were harvested individually, washed and re-suspended in FACS buffer. The cells were passed through a 70 μm cell strainer, and then subjected to another round of single cell isolation via FACS, just as described above. A final round of single cell sorting was performed 5 days later. 

#### 2.2.4. Viral Genomic DNA Extraction and PCRs

Wells containing fluorescent cells were selected for viral genomic DNA extraction using the MagVet™ Universal Isolation Kit (Thermo Fisher Scientific, Waltham, MA, USA), according to the manufacturer’s protocol, with minor modifications, including the use of a 40 μL instead of a 100 μL initial test sample, and the final elution in 60 μL of Elution Buffer NM6 instead of the recommended 80 μL. The lysed samples were extracted on the high-throughput KingFisher™ Flex Extraction System (Thermo Fisher Scientific, Waltham, MA, USA), using programmed script NM_LSI_RRC96. Extracted viral DNA was then subjected to PCR amplification to confirm the intended deletions, to verify the absence of carry-over parental virus and the presence of the fluorescent reporter gene (Table 1). The viral genomic DNA extraction and PCR amplifications were repeated after the limiting dilutions steps and the final virus propagation.

#### 2.2.5. Limiting Dilutions, Propagations and Titrations

Wells containing fluorescent cells with the desired gene-deleted virus were subjected to 2 rounds of limiting dilutions. The recombinant virus was then propagated in purified PBMs and titrated. Finally, viral genomic DNA flanking the whole recombination site was amplified to confirm the correct deletion. Briefly, viral DNA was extracted using the QIAamp Viral RNA Mini Kit (Qiagen, Hilden, Germany). The isolated DNA was amplified using proofreading Q5^®^ high-fidelity DNA polymerase (NEB, Hertfordshire, UK) (Table 2), and electrophoresed on 0.8% agarose gel.

## 3. Results and Discussion

FACS enables the sorting of a heterogeneous cell population into a relatively homogenous population, depending on their light scatters and fluorescent features. This technology has contributed greatly to understanding virus infections from identifying virus susceptible cells, characterizing viral protein expression, to defining host immune responses. Here, we combine homologous recombination and sorting of single cells infected with ASFV, expressing a fluorescent protein, to rapidly purify a genetically modified ASFV, Benin∆EP153R∆EP402R∆DP148R (Figure 1C). 

The parental Benin 97/1 isolate with DP148R gene deleted and replaced with GUS reporter has been previously described [18] (Figure 1B). The transfer plasmid p∆EP153R∆EP402R↔VP30mNG contains: (i) a fluorescent reporter gene: mNeonGreen (mNG) under the control of ASFV P30 promoter (5′-TTATTATTTTATAATTTTAAAATTGAATGGATTTTATTTTAAATATATCC); (ii) left flanking arm of EP153R (Benin 97/1 genomic positions 66,245–67,050); and (iii) right flanking arm of EP402R (Benin 97/1 genomic positions 68,776–69,580), all cloned into pUC57 vector (Figure 1A). The ASFV VP30 promoter was selected because P30 (CP204L) is expressed at both early and late stages of infection, and its promoter was shown to equally promote reporter gene expression in recombinant ASFV from early to late phases of infection [22]. Using this construct, 21bp of EP153R at the 5′ end remain in the recombinant virus, as it may contain the transcription termination signal for the adjacent EP152R gene [24,25].

Primary macrophages have a very low transfection efficiency [26,27], therefore, to increase the efficiency of recombination, the wild boar cell line, WSL-R, known to be ASFV susceptible [11,28,29], was used in the first step of producing the recombinant virus. WSL-R cells were infected with Benin∆DP148R, and then transfected with p∆EP153R∆EP402R↔VP30mNG. A centrifugation step at 600 *g* for 1h after both the infection and transfection steps was included to increase the recombination efficiency, as this was shown to enhance retroviral transductions [30] and improve liposome mediated transfections [31].

Cell preparation for live cell sorting is important to maintain cell integrity. To reduce the stress on cells, 48h post-infection and transfection, the following measures were implemented: (i) keeping cells cold throughout the experiment; (ii) the removal of supernatant and two washes to minimize extracellular virus and debris; (iii) minimal centrifugation speed (300 *g*) to sediment cells; and (iv) usage of FACS buffer that contains Ca^2+^/Mg^2+^ free PBS to reduce cell aggregation, EDTA to prevent cell adhesion and HEPES to improve buffer capacity during sorting. The cell morphology of infected-transfected cells was observed via the biparametric FSC vs. SSC profile (Figure 2 and Figure S1, Panel 1). The overall morphological differences between unmodified cells (Figure S1A, Panel 1), transfer plasmid transfected cells (Figure S1B, Panel 1), Benin∆DP148R (parental virus) and an additional fluorescent ASFV –infected cells (Figure S1C,D, Panel 1) were minimal. The FSC threshold was set to 5 K, so that debris would be detected and excluded during live cell sort. FACS of single mNeonGreen expressing cells was done with an 85 μm nozzle at 45 psi, to reduce additional stress on cells. The sorted single cell was eventually expected to release the recombinant virus into the fresh PBMs and, therefore, the initial gating included all possible cell fractions including potentially dead cells (Figure 2A, Panel 1).

Flow cytometric analysis at 48h post-infection/transfection showed that approximately 0.31% of the cells were positive for mNeonGreen, representing cells that are potentially infected with recombinant viruses (Figure 2A). Using the same method to prepare other recombinant viruses, we obtained approximately 0.1% to 3.0% of infected and transfected WSL-R cells. The mNeonGreen expressing population was then single cell sorted via FACS into individual wells of a 96-well plate containing purified PBMs. Despite sorting only single mNeonGreen positive cells, 5 days later, only 8.1% of wells when observed under a fluorescent microscope were positive for mNeonGreen. This phenomenon could either be associated with absence of homologous recombination in most of the cells or an abortive replication cycle in WSL-R cells. Hence, at least two or three steps of single cell isolation of mNeonGreen positive PBMs were necessary to obtain a pure recombinant virus culture with no parental virus contamination.

In the second round of FACS, cells were harvested from individual wells, in order to trace the virus population to the original single infected/transfected cell. The percentage of mNeonGreen expressing cells ranged from 0.71% to 4.45% of the total cell population (Figure 2B; Table S1). The relatively low percentage of mNeonGreen expressing cells could be due to the competing presence of the parental Benin∆DP148R. Single cells from the mNeonGreen positive population were sorted for a second time into plates containing purified PBMs. At five days post-infection, the percentage of wells that contained cells infected with the recombinant fluorescent virus increased to a range of 40.6–53.1% (Table S2). Cells from twelve mNeonGreen positive wells were then evaluated by flow cytometry, and the proportion of total cells infected with recombinant fluorescent virus ranged from 22.2–59.5% (Figure 2C, Table S1). This increase of approximately 10–80% in the proportion of PBMs expressing mNeonGreen, as compared to the previous round of FACS analysis, indicates increased purity with a lower contamination from parental virus. In order to further purify the recombinant Benin∆EP153R∆EP402R∆DP148R population, the final gating stringency was increased, so that only cells expressing mNeonGreen at a mean fluorescent intensity (MFI) above 5 × 10^3^ were single cell sorted into purified PBMs (Figure 2C, Panel 6). As expected, the number of sorted wells that were positive with mNeonGreen expressing PBMs, when monitored under a fluorescent microscope, dramatically increased to a range of 71.8–90.6% (Table S2). 

**Figure 3:**
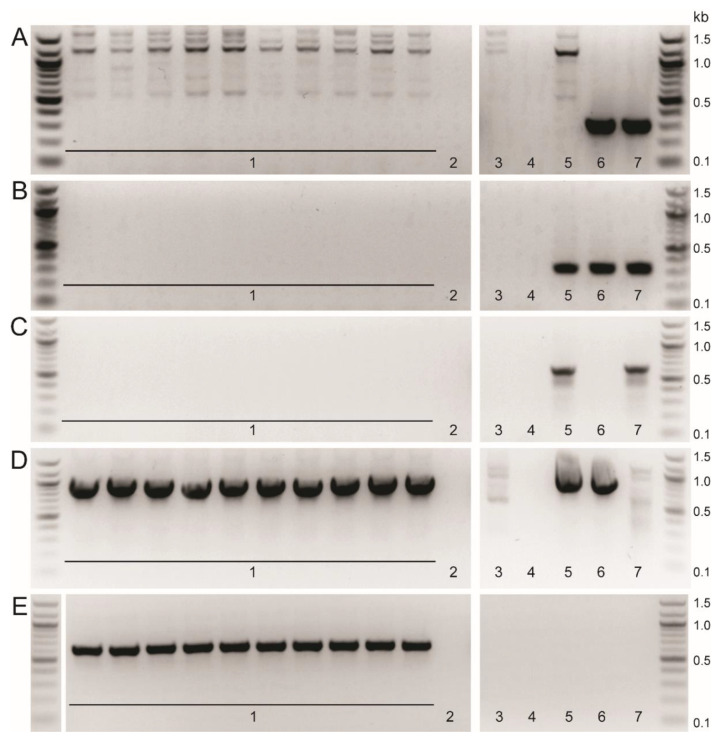
Representative PCR analyses of recombinant Benin∆EP153R∆EP402R∆DP148R showing the successful deletions of genes EP153R and EP402R. The extracted genomic viral DNA was subjected to PCR amplification and analysed by electrophoresis on 1.5% agarose gels. Panel depicts results from amplification of genes (**A**) DP148R (307 bp); (**B**) EP153R (297 bp); (**C**) EP402R (603 bp), (**D**) GUS (907 bp) and (**E**) mNeonGreen (634 bp). Lanes numbered **1** show analysis of samples from individual wells of purified Benin∆EP153R∆EP402R∆DP148R compared to **(2)** extraction negative control; **(3)** cell lysate control; **(4)** PCR negative control; **(5)** Benin∆DP148R control; **(6)** Benin∆EP402R control and **(7)** wildtype Benin 97/1 control.

The gradual increase in both the percentages of the total PBM population expressing mNeonGreen (1st round: 0.31%; 2nd round: 0.71–4.45%; 3rd round: 22.2–59.5%) and number of wells containing mNeonGreen positive cells (1st post-sort: 8.1%; 2nd post-sort: 40.6–53.1%; 3rd post-sort: 71.8–90.6%) over the rounds of single cell sorting reflects the enhanced purity of the Benin∆EP153R∆EP402R∆DP148R recombinant virus. PCR analysis showed that fragments corresponding to the EP153R and EP402R genes were not amplified, but the mNeonGreen reporter gene fragment was amplified from sixty percent of the wells containing mNeonGreen expressing cells. This indicates that a pure population of recombinant virus, without contaminating the parental virus, may be obtained by the third round of single cell sorting. However, two rounds of limiting dilution were performed to ensure the recombinant virus was completely free of parental virus. PCR analyses of DNA extracted from all wells containing cells infected with mNeonGreen expressing recombinant virus showed that, as expected, fragments corresponding to the deleted genes were not amplified, as seen in Figure 3A–C (A: DP148R; B: EP153R; C: EP402R). Fragments from the GUS and mNeonGreen reporter insertions were amplified (Figure 3D,E). It was noted that, following the first round of limiting dilution, a pure population of the recombinant virus is usually obtained. Therefore, we recommend an additional round of single cell isolation, or at least one round of limiting dilution, is included when this method is used, to ensure the recombinant ASFV is pure. Lastly, PCR amplifications encompassing the whole recombination site were undertaken with primers that anneal outside the cloned left and right flanking regions, in combination with primers that bind within the mNeonGreen gene (Table 2) or to the EP153R and EP402R genes (Table 1). As expected, the amplified viral DNA of approximately 1377 bp and 1395 bp was detected in the recombinant virus and absent in the wildtype Benin 97/1 isolate when the mNeonGreen primers were used (Figure S3A,B). The internal primers for genes EP153R and EP402R failed to detect amplicons in the recombinant virus, but amplified viral DNA from the wild type virus (Figure S3C,D). This confirms that the gene deletion and insertion of the reporter cassette had occurred correctly, as these primers also do not bind to the transfer plasmid. 

**Figure 4:**
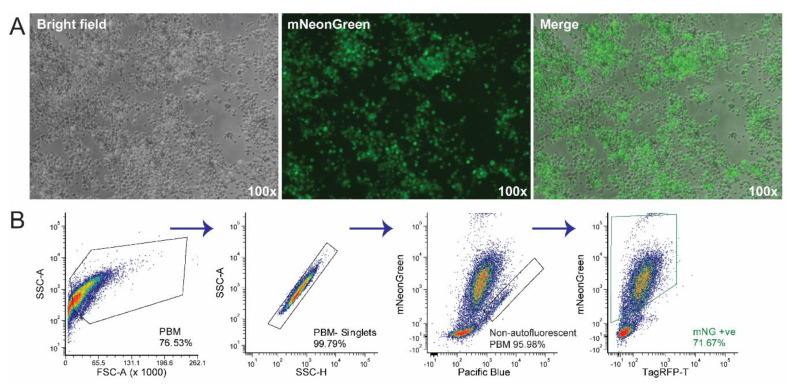
Primary bone marrow cells were infected with the produced recombinant ASFV Benin∆EP153R∆EP402R∆DP148R at MOI of 1. (**A**) The images were taken 5 days post-infection, showing the bright field image of purified PBMs, the mNeonGreen expressed by Benin∆EP153R∆EP402R∆DP148R—infected cells (green) and a merged image overlaying total purified PBMs with the infected PBMs. Magnifications are indicated in the images (100×). Images were taken using the EXi Blue fluorescence microscopy camera and QCapture Pro Version 7 (QImaging, Tucson, AZ, USA). (**B**) The same recombinant ASFV-infected PBMs were analysed by flow cytometry, and the biparametric profiles leading to gating of mNeonGreen positive cells are depicted. Gating strategy to identify infected cells; Panel 1: SSC-A vs FSC-A for total cells; Panel 2: SSC-A vs. SSC-H to obtain singlets; Panel 3: blue BP 530/30 nm-A vs. violet BP 450/40 nm-A to exclude autofluorescence cells, finally blue BP 530/30 nm-A vs. Yellow-Green (YG) BP 582/15 nm-A (Panel 4) to capture the mNeonGreen (mNG) positive cell subpopulation.

The final titre of the newly produced recombinant Benin∆EP153R∆EP402R∆DP148R (Figure 4) (1.68 × 10^8^ TCID_50_/mL) was not significantly different compared to the titres of Benin 97/1 isolate (1.78 × 10^7^ HAD_50_/mL) and the Benin∆DP148R (5.62 × 10^7^ HAD_50_/mL), as expected. Both EP153R and EP402R have been previously deleted from various ASFV isolate singly [32,33,34,35,36]. Benin∆EP153R∆EP402R∆DP148R is also non-haemadsorbing, compared to the virulent Benin 97/1 and attenuated Benin∆DP148R, which is expected, as EP402R (also known as CD2v) is responsible for haemadsorption [37] and ASFV isolates which have truncated or missing EP402R also cannot haemadsorb [38]. 

Using this single cell isolation method, it is feasible to use at least three different fluorescent markers in a single ASFV isolate, considering the filters available on the fluorescent microscope used to monitor microtiter plates. We have created several recombinant ASFV mutants with deleted genes replaced with TagRFP-T [39] or both TagRFP-T and mNeonGreen. With the presence of loxP flanking the reporters in the recombinant mutants, in the future, these fluorescent genes can be removed or swapped, allowing the researchers more flexibility. Most importantly, the time to obtain pure recombinant ASFV has been shortened to 1 to 1.5 months using this method, compared to the 3 to 4 months required for making a single gene deletion using purification via limiting dilution. In addition, the positive selection by FACS can greatly facilitate isolation of recombinant viruses that have a growth defect, compared to the parental virus.

## Figures and Tables

**Table 1:** Primers used in evaluating the absence or presence of target genes.

Gene Target	Primer Sequence (5′-3′)	Genome Position ^a^	Expected PCR Product Size (bp ^b^)
**DP148R**			
Forward	GTCCGCAACAAGGCTATTGAG	178,296–178,602	307
Reverse	GACGTTTACGCAGTGGGGC		
**EP153R**			
Forward	GATTGGAACTAATATGATAACTC	67,137–67,433	297
Reverse	TCACCACGTAATATTACCGT		
**EP402R**			
Forward	GTTCATGTTGTGGTCATAACATATC	67,763–68,365	603
Reverse	GAGATGGTTCATGTATGGAAGTG		
**GUS**			
Forward	GGTCCGTCCTGTAGAAACCCCAA	--	907
Reverse	GCCACGCAAGTCCGCATCTTCATG		
**mNeonGreen**			
Forward	TACACATCTTTGGCTCCATC	--	634
Reverse	CCCATCACATCGGTAAAGG		

^a^ genome position based on Benin 97/1 isolate. The -- indicates that primers do not bind anywhere in Benin 97/1. ^b^ bp—basepair.

**Table 2:** Primers used for sequencing the recombinant Benin∆EP153R∆EP402R∆DP148R.

Primer Name	Primer Sequence (5′-3′)	Genome Position ^a^
Del_EP153R_CD2v-SeqF1	CTTTGCCGTAGAAACAATACA	65,867
Del_EP153R_CD2v-SeqR3	TCAGGCTGTGTTCAATAA	69,978
mNG_SeqR	CCATGTCAAAGTCCACACCG	--
mNG_SeqF	CCAATGGCGGCTAACTATCTG	--

^a^ Genome position is based on Benin 97/1 isolate. The -- indicates that primers do not bind anywhere in Benin 97/1.

## Supplementary Materials

The following are available online at https://www.mdpi.com/1999-4915/12/6/615/s1. Figure S1: Infection-transfection controls. Figure S2: Controls for single cell purification via FACS. Figure S3: PCR analysis of recombinant Benin∆EP153R∆EP402R∆DP148R. Table S1: Percentage of mNeonGreen positive cells. Table S2: Percentage of mNeonGreen positive wells.

## References

[1] Netherton C.L., Connell S., Benfield C.T.O., Dixon L.K. (2019). The Genetics of Life and Death: Virus-Host Interactions Underpinning Resistance to African Swine Fever, a Viral Hemorrhagic Disease. Front. Genet..

[2] FAO Report on African Swine Fever (ASF) in Asia and the Pacific. Proceedings of the FAO Regional Conference for Asia and the Pacific.

[3] Arias M., De la Torre A., Dixon L., Gallardo C., Jori F., Laddomada A., Martins C., Parkhouse R.M., Revilla Y., Rodriguez F. (2017). Approaches and Perspectives for Development of African Swine Fever Virus Vaccines. Vaccines.

[4] Arias M., Jurado C., Gallardo C., Fernández-Pinero J., Sánchez-Vizcaíno J.M. (2018). Gaps in African swine fever: Analysis and priorities. Transbound. Emerg. Dis..

[5] Rodríguez J.M., Almazán F., Viñuela E., Rodriguez J.F. (1992). Genetic manipulation of African swine fever virus: Construction of recombinant viruses expressing the β-galactosidase gene. Virology.

[6] Gómez-Puertas P., Rodríguez F., Ortega A., Oviedo J.M., Alonso C., Escribano J.M. (1995). Improvement of African swine fever virus neutralization assay using recombinant viruses expressing chromogenic marker genes. J. Virol. Methods.

[7] Zsak L., Lu Z., Kutish G.F., Neilan J.G., Rock D.L. (1996). An African swine fever virus virulence-associated gene NL-S with similarity to the herpes simplex virus ICP34.5 gene. J. Virol..

[8] Neilan J.G., Lu Z., Kutish G.F., Zsak L., Burrage T.G., Borca M.V., Carrillo C., Rock D.L. (1997). A BIR Motif Containing Gene of African Swine Fever Virus, 4CL, Is Nonessential for Growthin Vitroand Viral Virulence. Virology.

[9] Afonso C.L., Zsak L., Carrillo C., Borca M.V., Rock D.L. (1998). African swine fever virus NL gene is not required for virus virulence. J. Gen. Virol..

[10] Hernaez B., Escribano J.M., Alonso C. (2006). Visualization of the African swine fever virus infection in living cells by incorporation into the virus particle of green fluorescent protein-p54 membrane protein chimera. Virology.

[11] Portugal R., Martins C., Keil G.M. (2012). Novel approach for the generation of recombinant African swine fever virus from a field isolate using GFP expression and 5-bromo-2′-deoxyuridine selection. J. Virol. Methods.

[12] O’Donnell V., Holinka L.G., Sanford B., Krug P.W., Carlson J., Pacheco J.M., Reese B., Risatti G.R., Gladue D.P., Borca M.V. (2016). African swine fever virus Georgia isolate harboring deletions of 9GL and MGF360/505 genes is highly attenuated in swine but does not confer protection against parental virus challenge. Virus Res..

[13] Borca M.V., O’Donnell V., Holinka L.G., Sanford B., Azzinaro P.A., Risatti G.R., Gladue D.P. (2017). Development of a fluorescent ASFV strain that retains the ability to cause disease in swine. Sci. Rep..

[14] Chen W., Zhao D., He X., Liu R., Wang Z., Zhang X., Li F., Shan D., Chen H., Zhang J. (2020). A seven-gene-deleted African swine fever virus is safe and effective as a live attenuated vaccine in pigs. Sci. China Life Sci..

[15] Abrams C.C., Dixon L.K. (2012). Sequential deletion of genes from the African swine fever virus genome using the cre/loxP recombination system. Virology.

[16] Borca M.V., Holinka L.G., Berggren K.A., Gladue D.P. (2018). CRISPR-Cas9, a tool to efficiently increase the development of recombinant African swine fever viruses. Sci. Rep..

[17] Chapman D.A.G., Darby A.C., Da Silva M., Upton C., Radford A.D., Dixon L.K. (2011). Genomic analysis of highly virulent Georgia 2007/1 isolate of African swine fever virus. Emerg. Infect. Dis..

[18] Reis A.L., Goatley L.C., Jabbar T., Sanchez-Cordon P.J., Netherton C.L., Chapman D.A.G., Dixon L.K. (2017). Deletion of the African Swine Fever Virus Gene DP148R Does Not Reduce Virus Replication in Culture but Reduces Virus Virulence in Pigs and Induces High Levels of Protection against Challenge. J. Virol..

[19] Enjuanes L., Carrascosa A.L., Moreno M.A., Viñuela E. (1976). Titration of African Swine Fever (ASF) Virus. J. Gen. Virol..

[20] Hierholzer J., Killington R. (1996). Virus isolation and quantitation. Virology Methods Manual.

[21] Shaner N.C., Lambert G.G., Chammas A., Ni Y., Cranfill P.J., Baird M.A., Sell B.R., Allen J.R., Day R.N., Israelsson M. (2013). A bright monomeric green fluorescent protein derived from Branchiostoma lanceolatum. Nat. Methods.

[22] Portugal R.S., Bauer A., Keil G.M. (2017). Selection of differently temporally regulated African swine fever virus promoters with variable expression activities and their application for transient and recombinant virus mediated gene expression. Virology.

[23] World Organisation for Animal Health (2019). Technical Disease Card for African Swine Fever.

[24] Almazán F., Rodríguez J.M., Andrés G., Pérez R., Viñuela E., Rodriguez J.F. (1992). Transcriptional analysis of multigene family 110 of African swine fever virus. J. Virol..

[25] Cackett G., Matelska D., Sýkora M., Portugal R., Malecki M., Bähler J., Dixon L., Werner F. (2020). The African Swine Fever Virus Transcriptome. J. Virol..

[26] Zhang X., Edwards J.P., Mosser D.M. (2009). The expression of exogenous genes in macrophages: Obstacles and opportunities. Macrophages and Dendritic Cells.

[27] Warwick C.A., Usachev Y.M., Kalyuzhny A.E. (2017). Culture, Transfection, and Immunocytochemical Analysis of Primary Macrophages. Signal Transduction Immunohistochemistry: Methods and Protocols.

[28] Hernaez B., Alonso C. (2010). Dynamin- and Clathrin-Dependent Endocytosis in African Swine Fever Virus Entry. J. Virol..

[29] de León P., Bustos M.J., Carrascosa A.L. (2013). Laboratory methods to study African swine fever virus. Virus Res..

[30] Kotani H., NewtonIII P.B., Zhang S., Chiang Y.L., Otto E., Weaver L., Blaese R.M., Anderson W.F., McGarrity G.J. (1994). Improved Methods of Retroviral Vector Transduction and Production for Gene Therapy. Hum. Gene Ther..

[31] Verma R.S., Giannola D., Shlomchik W., Emerson S.G. (1998). Increased Efficiency of Liposome-Mediated Transfection by Volume Reduction and Centrifugation. BioTechniques.

[32] Borca M.V., Carrillo C., Zsak L., Laegreid W.W., Kutish G.F., Neilan J.G., Burrage T.G., Rock D.L. (1998). Deletion of a CD2-Like Gene, *8-DR*, from African Swine Fever Virus Affects Viral Infection in Domestic Swine. J. Virol..

[33] Monteagudo P.L., Lacasta A., López E., Bosch L., Collado J., Pina-Pedrero S., Correa-Fiz F., Accensi F., Navas M.J., Vidal E. (2017). BA71ΔCD2: A New Recombinant Live Attenuated African Swine Fever Virus with Cross-Protective Capabilities. J. Virol..

[34] Borca M.V., O’Donnell V., Holinka L.G., Risatti G.R., Ramirez-Medina E., Vuono E.A., Shi J., Pruitt S., Rai A., Silva E. (2020). Deletion of CD2-like gene from the genome of African swine fever virus strain Georgia does not attenuate virulence in swine. Sci. Rep..

[35] Galindo I., Almazán F., Bustos M.J., Viñuela E., Carrascosa A.L. (2000). African Swine Fever Virus EP153R Open Reading Frame Encodes a Glycoprotein Involved in the Hemadsorption of Infected Cells. Virology.

[36] Hurtado C., Bustos M.J., Granja A.G., de León P., Sabina P., López-Viñas E., Gómez-Puertas P., Revilla Y., Carrascosa A.L. (2011). The African swine fever virus lectin EP153R modulates the surface membrane expression of MHC class I antigens. Arch. Virol..

[37] Rodríguez J.M., Yáñez R.J., Almazán F., Viñuela E., Rodriguez J.F. (1993). African swine fever virus encodes a CD2 homolog responsible for the adhesion of erythrocytes to infected cells. J. Virol..

[38] King K., Chapman D., Argilaguet J.M., Fishbourne E., Hutet E., Cariolet R., Hutchings G., Oura C.A.L., Netherton C.L., Moffat K. (2011). Protection of European domestic pigs from virulent African isolates of African swine fever virus by experimental immunisation. Vaccine.

[39] Shaner N.C., Lin M.Z., McKeown M.R., Steinbach P.A., Hazelwood K.L., Davidson M.W., Tsien R.Y. (2008). Improving the photostability of bright monomeric orange and red fluorescent proteins. Nat. Methods.

