# Supplementary material for: Production of Recombinant African Swine Fever Viruses: Speeding Up the Process
Source: Viruses. 2020 Jun 5;12(6):615. doi: 10.3390/v12060615 (PMC7354605; doi:10.3390/v12060615)
Supplement: Supplementary file 1 [file viruses-12-00615-s001.pdf]

Supplementary Materials

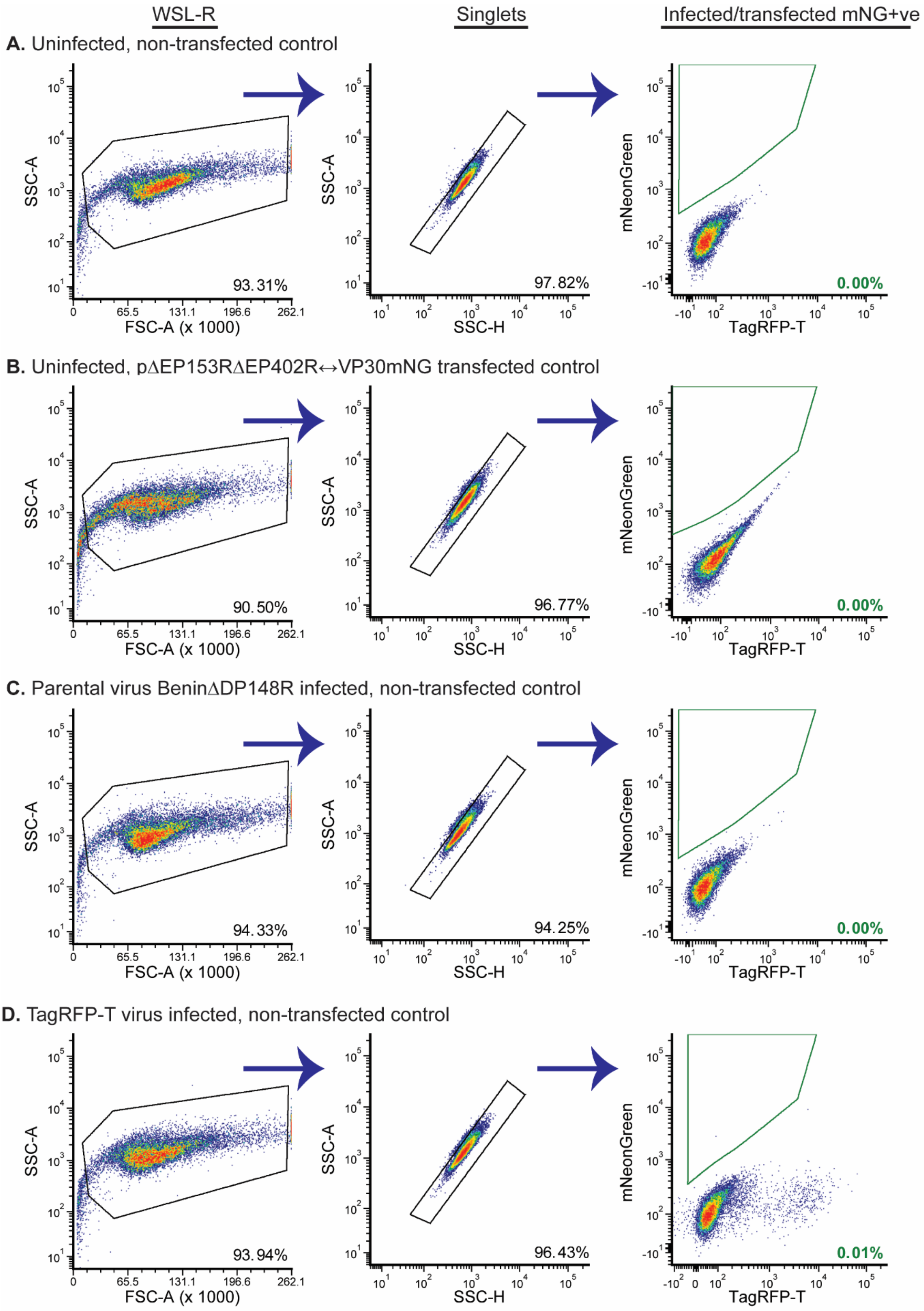

Figure S1: Infection-transfection controls. Biparametric flow cytometry profiles for controls during the infection-transfection step of single cell isolation where Panel 1 – SSC-A vs FSC-A for total cells; Panel 2 – SSC-A vs. SSC-H to obtain singlets; Panel 3 – blue BP 530/30nm-A vs. Yellow-Green (YG) BP 582/15nm-A to capture the mNeonGreen (mNG) positive cell subpopulation. Gating for the final infected-transfected WSL-R expressing mNeonGreen (Figure 2A) was based on the following controls: (**A**) uninfected, non-transfected cells; (**B**) uninfected, transfer plasmid transfected cells; (**C**) parental virus infected, non-transfected cells; (**D**) alternative fluorescent virus (red fluorescent) infected, non-transfected cells.

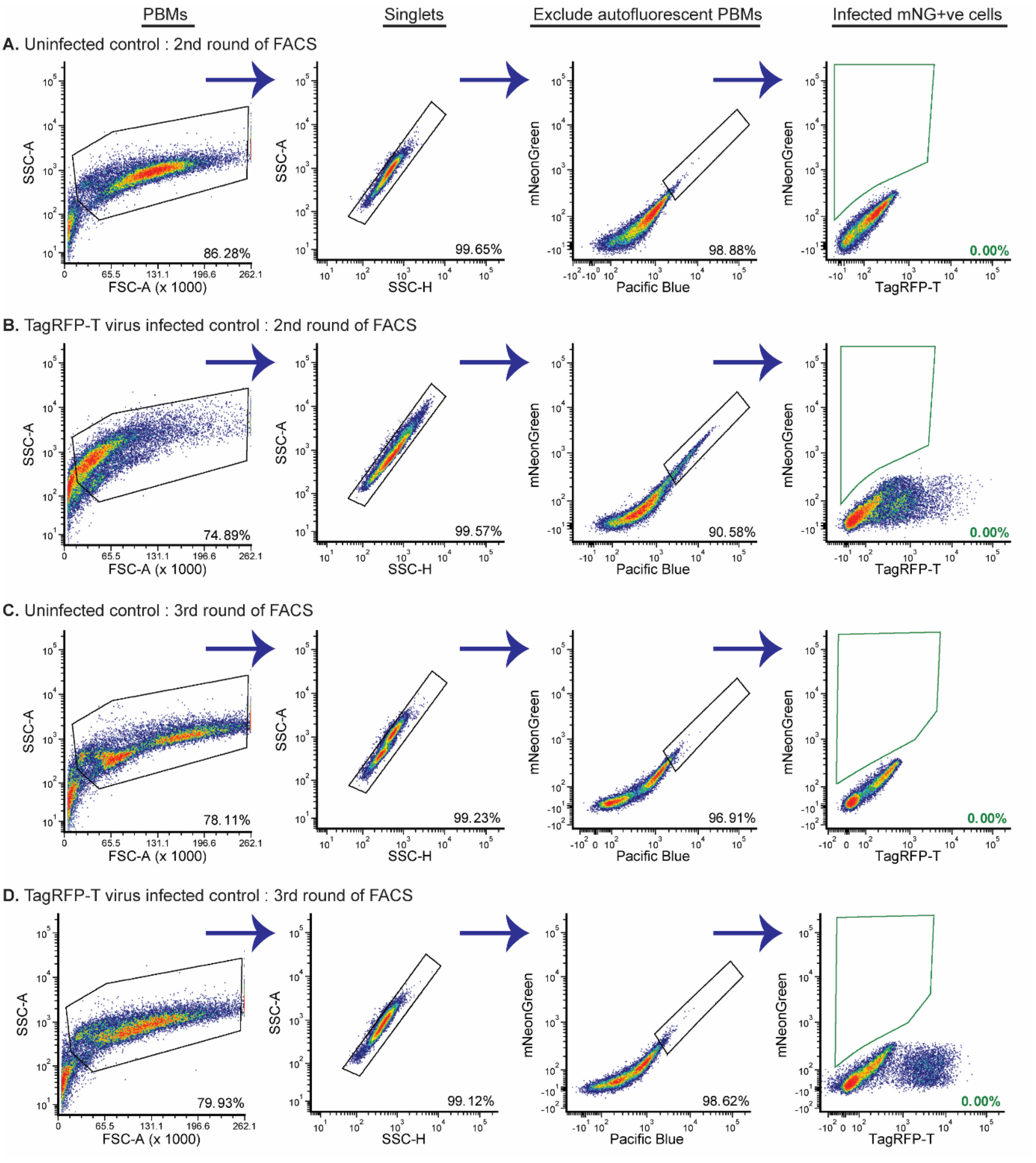

Figure S2: Controls for single cell purification via FACS. Biparametric flow cytometry profiles for controls following the first single cell sorting. The panels include Panel 1 – SSC-A vs FSC-A for total cells; Panel 2 – SSC-A vs. SSC-H to obtain singlets; Panel 3- blue BP 530/30nm-A vs. violet BP 450/40nm-A to exclude autofluorescence cells and Panel 4 – blue BP 530/30nm-A vs. Yellow-Green (YG) BP 582/15nm-A to capture the mNeonGreen (mNG) positive cell subpopulation. Gating for the final BeninΔEP153RΔEP402RΔDP148R infected cells (Figure 2B, C) was based on the following controls (**A,C**) uninfected purified PBMs and (**B, D**) an additional fluorescent virus infected purified PBMs.

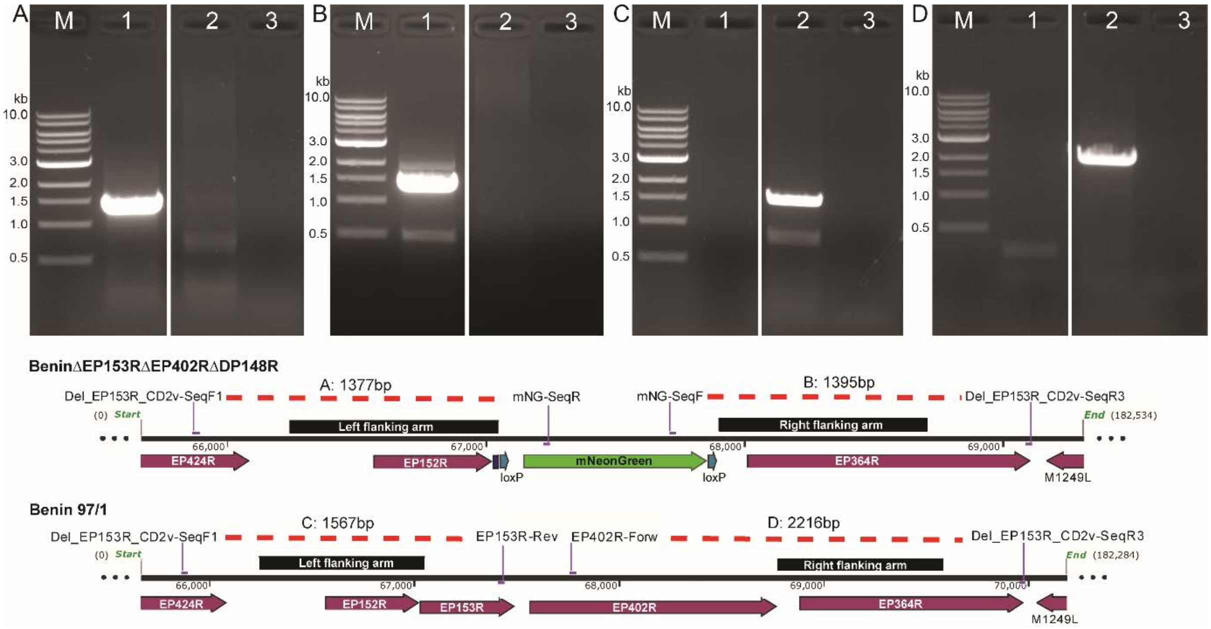

Figure S3: PCR analysis of recombinant BeninΔEP153RΔEP402RΔDP148R. The extracted genomic viral DNA was subjected to PCR amplification with a combination of primers from Tables 1 and 2. The PCR products were analysed by electrophoresis on 0.8% agarose gels. Lanes (M) shows the 1kb DNA Ladder (NEB); (1) represents BeninΔEP153RΔEP402RΔDP148R; (2) shows Benin 97/1 isolate; and (3) PCR negative control. (A) PCR was performed using primers Del_EP153R_CD2v-SeqF1 and mNG-SeqR, which amplified an expected product of approximately 1377bp in the recombinant virus and none in the wildtype Benin 97/1 isolate. Likewise, using primers Del_EP153R_CD2v-SeqR3 and mNG-SeqF, a product of approximately 1395bp was detected in the recombinant virus and not in the Benin 97/1 isolate (B). To further substantiate, the primers located outside the flanking arms were amplified with internal primers for genes EP153R (C) and EP402R (D), which failed to produce any amplicons in the recombinant virus, but amplified as expected products of approximately 1567bp (C) and 2216bp (D) in the Benin 97/1 isolate. The diagram shown below the gel electrophoresis images, shows where the respective primers are located and the PCR product size estimated from both the BeninΔEP153RΔEP402RΔDP148R and Benin 97/1 ASFV isolates.

Table S1: Percentage of mNeonGreen positive cells. Percentage of cells infected with ASFV BeninΔEP153RΔEP402RΔDP148R expressing mNeonGreen reporter over the total number of cells analysed via flow cytometer.

Flow cytometry	mNG +ve cells/ total cells (%)
1st round: WSL-R	
DEC	0.31
2nd round: purified PBM	
DEC1	3.15
DEC2	4.45
DEC3	2.72
DEC4	0.71
DEC5	1.63
DEC6	0.99
3rd round: purified PBM	
DEC1.1	30.63
DEC1.2	59.47
DEC2.1	51.00
DEC2.2	54.04
DEC3.1	22.18
DEC3.2	43.16
DEC4.1	57.35
DEC4.2	48.33
DEC5.1	55.40
DEC5.2	54.71
DEC6.1	35.33
DEC6.2	23.23

mNG – mNeonGreen ; +ve – positive; WSL-R – wild boar cells; PBM: porcine bone marrow cells

DEC – BeninΔEP153RΔEP402RΔDP148R. The numbers after DEC refers to the individual well analysed.

Table S2: Percentage of mNeonGreen positive wells. Percentage of wells containing cells that were infected with recombinant ASFV BeninΔEP153RΔEP402RΔDP148R expressing mNeonGreen after every cycle of single cell isolation when monitored under fluorescent microscope.

Fluorescent microscopy	mNG +ve wells (%)
Post 1st round sorting	
DEC	8.1
Post 2nd round sorting	
DEC1	46.9
DEC2	43.8
DEC3	46.9
DEC4	40.6
DEC5	53.1
DEC6	40.6
Post 3rd round sorting	
DEC2.1 (1)	71.8
DEC2.1 (2)	84.4
DEC6.1	90.6

mNG – mNeonGreen ; +ve – positive;

DEC – BeninΔEP153RΔEP402RΔDP148R. The numbers after DEC refers to the single sorted plates checked under a fluorescent microscope.
